# The Efficacy and Safety of Somatostatin Analog after Axillary Node Dissection in Breast Cancer: A Systematic Review and Meta-analysis

**DOI:** 10.31662/jmaj.2022-0219

**Published:** 2023-05-22

**Authors:** Satsuki Hirono, Jun Watanabe, Atsushi Miki, Mikio Shiozawa, Naohiro Sata

**Affiliations:** 1 Department of Surgery, Division of Gastroenterological, General and Transplant Surgery, Jichi Medical University, Shimotsuke, Japan; 2 Division of Community and Family Medicine, Jichi Medical University, Shimotsuke, Japan

**Keywords:** breast cancer, axillary lymphadenectomy, octreotide, seroma, somatostatin

## Abstract

**Background:**

Somatostatin analogs are expected to reduce lymphatic leakage. However, whether they can be used after axillary lymphadenectomy is unclear. This study aimed to assess the efficacy and safety of somatostatin analogs in axillary lymphadenectomy for breast cancer patients.

**Methods:**

We performed a random-effects meta-analysis by searching electronic databases for randomized trials and trial registries until June 2022. The primary outcomes were the volume of drained fluid, the duration of drainage, and seroma incidence. Bias was assessed using the Cochrane Collaboration’s tool and the Grading of Recommendations, Assessment, Development, and Evaluations approach.

**Results:**

Six trials (738 participants) and one protocol without results were included. Somatostatin analogs may reduce the volume of drained fluid (mean difference = −22.07 mL, 95% confidence interval [CI] = −42.09 to −2.05; I^2^ = 56%) while resulting in a slight-to-no difference in the duration of drainage (mean difference = −0.48 days, 95% CI = −1.43 to 0.46; I^2^ = 87%) and seroma incidence (risk ratio = 0.91, 95% CI = 0.61-1.34; I^2^ = 55%). The certainty of the evidence was low.

**Conclusions:**

There was limited evidence supporting somatostatin analogs for lymphorrhea after axillary lymphadenectomy. Multicenter randomized controlled trials are needed to confirm the efficacy and safety of somatostatin analogs after axillary lymphadenectomy.

## Introduction

Invasive breast cancer is diagnosed in 2.3 million women worldwide and causes 685,000 deaths. In the past five years, 7.8 million women diagnosed with breast cancer survived, making it the most prevalent cancer in the world ^(1)^. Axillary dissection is commonly performed in patients with breast cancer with axillary lymph node metastasis and is associated with the development of postoperative lesions, such as seroma and hematoma, and infection ^(2), (3)^. Seromas require medical intervention, increase the risk of infection, prolong hospital stay, and may delay the start of chemotherapy, radiation therapy, or both ^(4)^. Seroma formation is a serious and disabling complication of axillary lymph node dissection, but there is currently no effective treatment ^(5)^.

Octreotide, a somatostatin analog, has been successfully used in the medical management of postoperative gastrointestinal and pancreatic fistulas ^(6)^. Octreotide has also been used in an attempt to control lymphatic leakage in thoracic duct injuries after radical neck and pelvic lymph node dissection ^(7), (8)^. Other somatostatin analogs, namely, pasireotide and lanreotide, also show a higher affinity than that of octreotide and are expected to significantly reduce lymphatic leakage ^(9), (10)^. However, whether somatostatin analogs are effective after axillary node dissection in breast cancer is unclear.

The purpose of this study was to assess the efficacy and safety of somatostatin analogs in axillary lymphadenectomy for breast cancer patients.

## Materials and Methods

We followed the Preferred Reporting Items for Systematic Review and Meta-Analysis 2020 (PRISMA-2020) ^(11)^. We registered this review protocol in the Open Science Forum (doi: 10.17605/OSF.IO/REH67) and in PROSPERO (CRD42022341543).

### Study selection

We included randomized controlled trials (RCTs) that assessed the efficacy and safety of somatostatin analogs after axillary node dissection in breast cancer. We did not apply language or country restrictions. We included all papers, such as published and unpublished articles, conference abstracts, and letters. We excluded non-RCTs. We did not exclude studies based on the observation period or publication year. Inclusion criteria were patients aged over 18 years with breast carcinoma. The exclusion criterion was patients with contraindications to somatostatin analogs. The intervention was administration of a somatostatin analog, including somatostatin, octreotide, lanreotide, pasireotide, or vapreotide. The control was no treatment or placebo administration. The primary outcomes were the volume of drained fluid (mL), the duration of drainage (days), and seroma incidence. Seroma incidence was defined as the number of patients with seroma divided by the total number of patients. The secondary outcomes were hematoma, surgical site infection, length of hospital stay, and all adverse events.

We searched the following databases: MEDLINE (PubMed), the Cochrane Central Register of Controlled Trials (Cochrane Library), and EMBASE (Dialog) (Supplementary 1). We also searched the following databases for ongoing or unpublished trials: the World Health Organization International Clinical Trials Platform Search Portal (ICTRP) and ClinicalTrials.gov (Supplementary 2). We checked the reference lists of studies, including international guidelines ^(2), (3)^, as well as the reference lists of eligible studies and articles that cited eligible studies using citationchaser ^(12)^. We asked the authors of original studies for unpublished or additional data.

### Data collection and analysis

Two independent reviewers (SH and JW) screened titles and abstracts, followed by an assessment of eligibility based on the full texts. We contacted original authors if relevant data were missing. Two reviewers (SH and JW) performed an independent data extraction of the information on the study design, study population, interventions, and outcomes. Two reviewers (SH and JW) evaluated the risk of bias independently using the Risk of Bias 2 tool ^(13)^. Disagreements between the two reviewers were discussed, and if they failed to reach a consensus, a third reviewer (AM) acted as an arbiter.

### Data synthesis and analyses

We pooled the relative risk ratios (RR) and 95% confidence intervals (CIs) for the following binary variables: seroma incidence, hematoma incidence, and surgical site infection. We pooled the mean differences (MD) and 95% CIs for the following continuous variables: volume of drained fluid, duration of drainage, and lengths of hospital stay. Meta-analysis was performed using Review Manager software (RevMan 5.4). We used a random-effects model. We summarized adverse events based on the definition in the original article, but we did not perform meta-analysis. We performed the intention-to-treat analysis for all dichotomous data, when possible. For continuous data, we did not impute missing data based on the recommendation by the Cochrane handbook ^(13)^. We performed a meta-analysis of the available data in the original study.

We evaluated statistical heterogeneity by a visual inspection of the forest plots and by calculating the I^2^ statistic (I^2^ values of 0%-40%: might not be important; 30%-60%: may represent moderate heterogeneity; 50%-90%: may represent substantial heterogeneity; and 75%-100%: considerable heterogeneity) based on the Cochrane handbook ^(13)^. When there was substantial heterogeneity (I^2^ > 50%), we assessed the reason for the heterogeneity using the following subgroup analysis: surgery type (mastectomy, breast-conserving surgery, or others) and somatostatin analog administered (somatostatin, octreotide, lanreotide, pasireotide, or vapreotide). However, the prespecified subgroup analysis of the type of surgery could not be performed because all studies included only mastectomies. The following sensitivity analyses were planned: exclusion of studies with imputed statistics and exclusion of studies in which participants had missing data. The prespecified sensitivity analysis of the exclusion of studies using imputed statistics was not performed because there were no studies using imputed statistics.

We searched the clinical trial registry system (ClinicalTrials.gov and ICTRP) and performed an extensive literature search for unpublished trials. To assess the outcome reporting bias, we compared the outcomes defined in the trial protocols with the outcomes reported in the publications. On the basis of the Cochrane handbook guidelines, we did not conduct the funnel plot analysis and Egger test because we found less than 10 trials ^(13)^. Summary of findings tables were prepared for the outcomes based on the Cochrane handbook ^(13)^. We adopted corresponding risks from the median of the included trials. We included grading to evaluate the quality of the evidence based on the Grading of Recommendations, Assessment, Development, and Evaluations (GRADE) approach for each summary of findings table ^(14)^.

## Results

**Figure 1.**
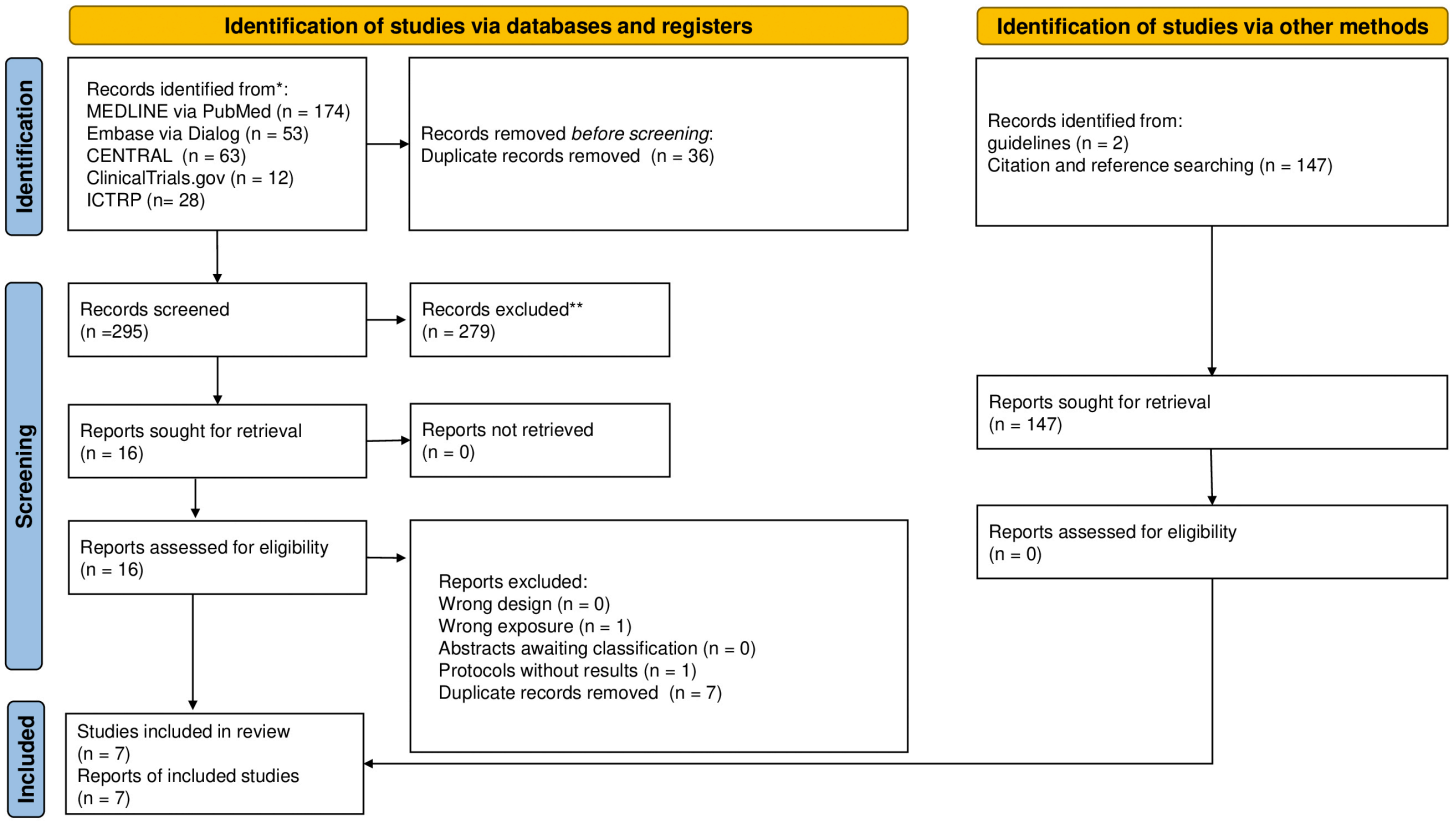
Flow of the study selection process.

Figure 1 shows the literature search process. A total of 331 records were identified through our database search. After the removal of duplicate publications, 295 records were screened and 16 full-text documents were checked. The following nine studies were then excluded: seven studies with duplicate records, one comparative study of the long- versus short-term use of octreotide ^(15)^, and one protocol without results ^(16)^. Ultimately, seven studies with 738 participants were considered eligible for inclusion ^(17), (18), (19), (20), (21), (22), (23)^. No additional articles that met the inclusion criteria were identified from the reference lists of studies.

Table 1 summarizes the characteristics of the seven eligible studies. In all eligible studies, the surgery was mastectomy and axillary lymph node dissection ^(17), (18), (19), (20), (21), (22), (23)^. The interventions were the administration of octreotide in five studies^(17), (18), (19), (22), (23)^, lanreotide in one study ^(20)^, and pasireotide in one study ^(21)^. Table 2 shows the study quality for the eligibility studies using the Risk of Bias 2 tool. The overall risk of bias ranged from “low” to “some concerns” because of the lack of information on concealment methods and preregistration protocols (Supplementary 3).

**Table 1.** Summary of the Characteristics of the Eligibility Studies.

Authors [reference]	Year	Country	Subjects (intervention/ control)	Age (years) (intervention/ control)	Surgery type	Intervention (somatostatin analog)	Control	Published or unpublished
Carcoforo ^(17)^	2003	Italy	125/136	63/63	Mastectomy	Octreotide	Standard treatment	Published
Mahmoud ^(18)^	2007	Egypt	30/20	47/49	Mastectomy	Octreotide	Standard treatment	Published
Chemi SpA ^(19)^	2012	Italy	86/28	56/54	Mastectomy	Octreotide	Placebo	Unpublished
Gauthier ^(20)^	2012	France	72/73	56/59	Mastectomy	Lanreotide	Placebo	Published
Chéreau ^(21)^	2016	France	42/48	55/52	Mastectomy	Pasireotide	Placebo	Unpublished
Chemi SpA ^(22)^	2020	Italy	24/24	NR/NR	Mastectomy	Octreotide	Placebo	Published
Prajapati ^(23)^	2021	India	15/15	48/45	Mastectomy	Octreotide	Standard treatment	Published

NR, not reported

**Table 2:** Quality scores of the Eligibility Studies for the Drained Fluid Volume.

Authors [reference]	Risk of bias 2 tool assessment					
	Bias arising from the randomization process	Bias due to deviations from intended interventions	Bias due to missing outcome data	Bias in the measurement of the outcome	Bias in the selection of the reported results	Overall risk of bias
Carcoforo ^(17)^	Some concerns	Low	Low	Low	Some concerns	Some concerns
Mahmoud ^(18)^	Some concerns	Low	Low	Some concerns	Some concerns	Some concerns
Gauthier ^(20)^	Some concerns	Low	Low	Low	Some concerns	Some concerns
Chéreau ^(21)^	Low	Low	Low	Low	Low	Low
Prajapati ^(23)^	Low	Low	Low	Low	Low	Low

### Outcomes

Table 3 summarizes the findings using the GRADE approach. The certainty of the evidence was low to very low because of the inconsistency due to substantial heterogeneity, imprecision due to the small sample size, and indirectness.

**Table 3.** Summary of Findings.

The efficacy and safety of somatostatin analog after axillary node dissection						
Patient or population: Adults with breast cancer with axillary lymph node metastasis; Setting: Inpatients; Intervention: Somatostatin analog; Comparison: Placebo or standard treatment						
Outcomes	Anticipated absolute effects^*^ (95% CI)		Relative effect (95% CI)	Patients (studies)	Certainty of the evidence (GRADE)	Comments
	Risk with control	Risk with somatostatin analog				
Drained fluid volume	Median volume of drained fluid = 145 mL	MD −22.1 mL (−42.1 to −2.1)	-	576 (5 RCTs)	Low^a,b^	Somatostatin analog reduced the drained fluid volume
Drainage duration	Median duration of drainage = 4.5 days	MD −0.48 days (−1.43 to 0.46)	-	685 (6 RCTs)	Low^a,b^	Somatostatin analog resulted in little to no difference in the drainage duration
Seroma	459 per 1000	418 per 1000 (280 to 615)	RR 0.91 (0.61 to 1.34)	311 (4 RCTs)	Low^a,b^	Somatostatin analog resulted in little to no difference in seroma
Hematoma	8 per 1000	4 per 1000 (1 to 28)	RR 0.53 (0.08 to 3.53)	253 (3 RCTs)	Low^b,c^	Somatostatin analog resulted in little to no difference in hematoma
Surgical site infection	31 per 1000	19 per 1000 (8 to 44)	RR 0.62 (0.27 to 1.41)	598 (5 RCTs)	Low^b,c^	Somatostatin analog resulted in little to no difference in surgical site infection
Length of hospitalization	Median length of hospitalization = 5 days	MD −0.79 SD (−1.30 to −0.27)	-	526 (4 RCTs)	Low^a,b^	Somatostatin analog reduced the length of hospitalization

CI, confidence interval; MD, mean difference; RR, risk ratio. *The risk of the intervention group (and its 95% CI) is based on the assumed risk of the comparison group and the relative effect of the intervention (and its 95% CI). GRADE Working Group grades of evidence: high certainty, we are very confident that the true effect is close to that of the estimated effect; moderate certainty, we are moderately confident in the estimated effect and that the true effect is likely close to the estimated effect, but there is a possibility that it is substantially different; low certainty: our confidence in the estimated effect is limited and the true effect may be substantially different from the estimated effect; very low certainty, we have very little confidence in the estimated effect and believe that the true effect is likely to be substantially different from the estimated effect. ^a^Downgraded because of inconsistency caused by substantial heterogeneity. ^b^Downgraded because of imprecision caused by the small sample size. ^c^Downgraded because of indirectness.

### Primary outcomes

**Figure 2.**
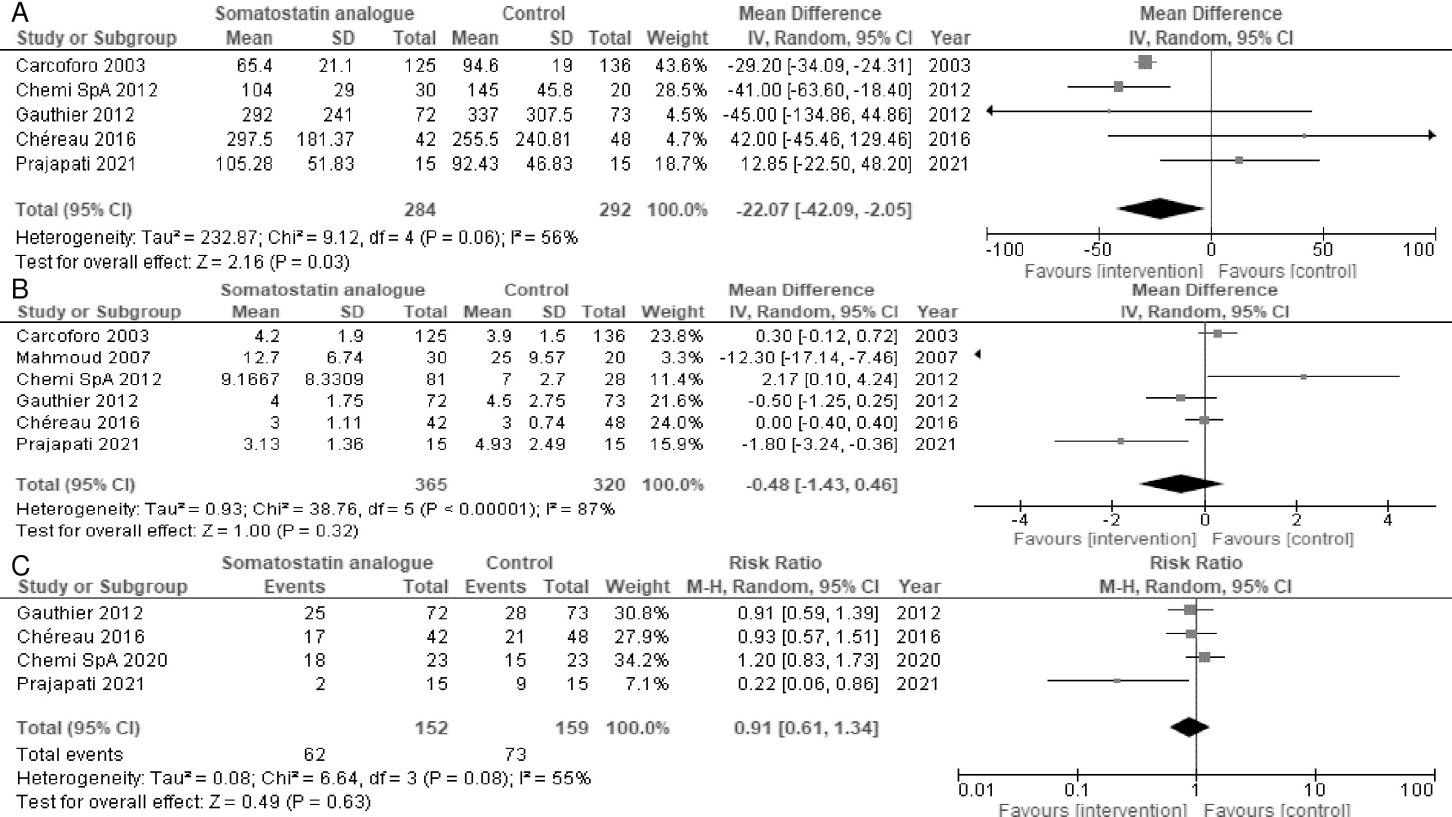
Forest plot of A) the volume of drained fluid (mL), B) the duration of drainage (days), and C) seroma incidence.

Five studies reported the volume of drained fluid. Somatostatin analogs may reduce the volume of drained fluid (MD = −22.07 mL, 95% CI = −42.09 to −2.05; I^2^ = 56%) (Figure 2A) ^(17), (18), (20), (21), (23)^. Six and four studies reported the duration of drainage and seroma incidence, respectively. Somatostatin analogs resulted in a slight-to-no difference in the duration of drainage (MD = −0.48 days, 95% CI = −1.43 to 0.46; I^2^ = 87%) (Figure 2B) ^(17), (18), (19), (20), (21), (23)^ and seroma incidence (RR = 0.91, 95% CI = 0.61-1.34; I^2^ = 55%) (Figure 2C) ^(20), (21), (22), (23)^. Subgroup analyses were performed for primary outcomes because substantial heterogeneity was identified. However, the subgroup analyses of the somatostatin analog types showed no significant differences (test for subgroup differences, p > 0.05) (Appendix Figure 1A−1C). The sensitivity analysis of only participants with complete data for the volume of drainage was not consistent with the primary findings. Somatostatin analogs resulted in a slight-to-no difference in the volume of drainage (MD = −11.97 mL, 95% CI = −52.50 to 28.56; I^2^ = 81%) (Appendix Figure 2A). The sensitivity of only the participants with complete data for the duration of drainage and seroma incidence was consistent with the primary findings (Appendix Figure 2B, 2C).

### Secondary outcomes

**Figure 3.**
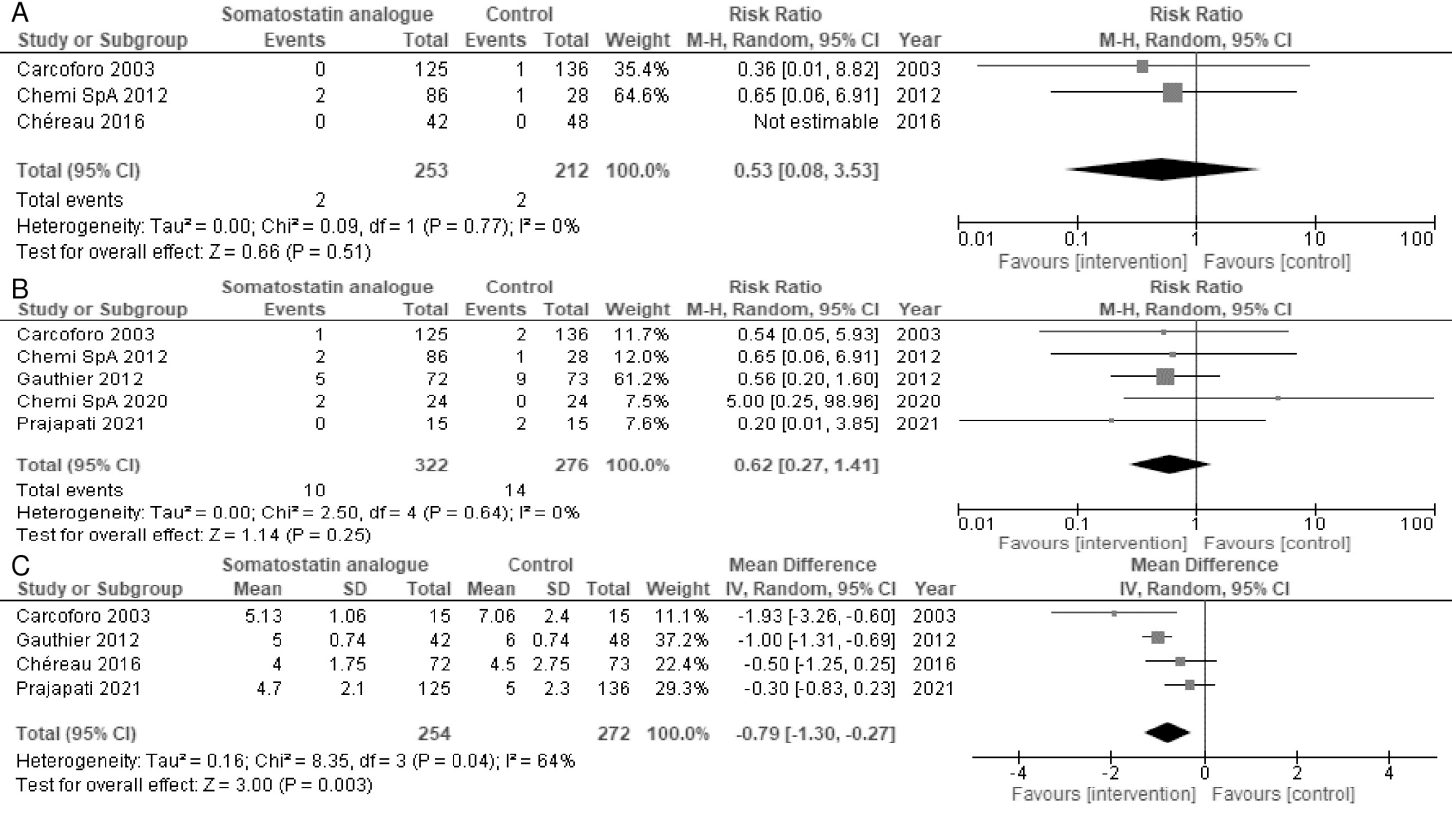
Forest plot of A) hematoma incidence, B) surgical site infections, C) length of hospital stay.

Three and five studies reported the duration of hematoma and surgical site infections, respectively. Somatostatin analogs resulted in a slight-to-no difference in hematoma incidence (RR = 0.53, 95% CI = 0.08-3.53; I^2^ = 0%) (Figure 3A) and surgical site infections (RR = 0.62, 95% CI = 0.27-1.41; I^2^ = 0%) (Figure 3B). Somatostatin analogs may reduce the length of hospital stay (MD = −0.79 days, 95% CI = −1.30 to −0.27; I^2^ = 64%) (Figure 3C). The subgroup analyses of the length of hospital stay by the somatostatin analog type did not differ significantly (test for subgroup differences, p = 0.48) (Appendix Figure 3). The sensitivity analysis of only the participants with complete data for hematoma incidence and surgical site infections did not differ significantly (Appendix Figure 4A, 4B), whereas the sensitivity analysis of only the participants with complete data for the length of hospital stay was not consistent with the primary results (MD = −1.0 days, 95% CI = −2.58 to 0.58; I^2^ = 64%) (Appendix Figure 4C).

### All adverse events

Four studies reported adverse events ^(19), (21), (22), (23)^. Of the four studies ^(19), (21), (22), (23)^, two reported serious adverse events ^(19), (22)^. There were 1/24 (4.2%) and 4/86 (4.7%) serious adverse events in the somatostatin analog group and 1/24 (4.2%) and 3/28 (10.7%) serious adverse events in the control group in the two studies ^(19), (22)^. The most common adverse events were vomiting (6%), diarrhea (13%), and injection site pain (10%) ^(19), (22)^.

## Discussion

This systematic review and meta-analysis that included 7 RCTs with 738 participants demonstrated that somatostatin analogs may reduce the volume of drained fluid but may result in a slight-to-no difference in the reduction of the duration of drainage and seroma incidence. Our findings indicate that the evidence is very uncertain regarding the efficacy of somatostatin analogs for lymphatic leakage in axillary lymphadenectomy for breast cancer patients. This study is the first systematic review and meta-analysis focusing on the efficacy and safety of somatostatin analogs in axillary lymphadenectomy for breast cancer patients.

The results of this systematic review and meta-analysis suggest that on the basis of the current evidence, somatostatin analogs cannot be confirmed as effective or safe. This conclusion is in contrast to the conclusion drawn in the first systematic review that argued that somatostatin analogs are safe alternatives that produce results comparable to those of surgery ^(24)^. One reason for the discrepancy may be related to the difference in the number of studies included. The previous study included only one study ^(24)^, whereas seven studies were included in the present review. This suggests that this review involves a more comprehensive literature search. In an earlier systematic review, neither the modification of the surgical technique nor the application of the medical treatment was effective in preventing lymphatic leakage ^(25)^. However, in an earlier review, clinical heterogeneity was extremely high because various medical treatments were meta-analyzed together. The present review focuses on somatostatin analogs and includes an assessment of the certainty of the evidence for each outcome and a rationale for the evaluation based on the GRADE approach.

In this review, somatostatin analogs resulted in little-to-no difference in the incidence of hematoma and surgical site infections. In a previous systematic review, 14 risk factors for breast surgical site infections were identified, including aging, hypertension, a high body mass index, hematoma, seroma, and postoperative drains ^(26)^. Because only RCTs were included in this review, patients’ backgrounds did not differ between the somatostatin analog and control groups, and the results for hematoma duration and seroma incidence were not significantly different. Thus, the risk factors for surgical site infections in the two groups were equal. Therefore, the present results are consistent with those of a previous systematic review.

In the present review, somatostatin analogs may reduce the length of hospital stay by 0.8 days. In a Cochrane systematic review, wound drainage after axillary dissection for breast carcinoma reduced seroma formation but prolonged the length of hospital stay by 1.5 days ^(5)^. However, wound drainage after axillary dissection decreases seroma formation, and wound drainage is commonly used in clinical practice. In the present review, the overall quality of the data was poor and the sensitivity analysis did not agree with the initial results. Therefore, using somatostatin analogs in real-world clinical practice to reduce the length of hospital stay should be performed with caution.

In this study, adverse events were comparable between the somatostatin analog and control groups. In a previous systematic review of somatostatin analogs, adverse events occurred in 18% of patients receiving somatostatin analog therapy and the most common adverse events were loose stools (3%) and administration site reactions (2%). Our study corroborates these prior studies and extends them by demonstrating that adverse events are comparable to those in the control group.

Several limitations associated with the present study warrant mention. First, this review included only seven RCTs. Although the sample size was limited, this study was comprehensive in presenting the current evidence, including two unpublished datasets ^(19), (22)^. Second, the studies included in this review varied with respect to the doses and types of somatostatin analogs. Further studies are needed to increase the certainty and generalizability of the evidence.

In conclusion, somatostatin analogs may reduce the volume of drained fluid, but it is unclear whether they reduce the duration of drainage and seroma incidence. Given the low certainty of the evidence for the efficacy of somatostatin analogs in preventing lymphatic leakage, the routine use of somatostatin analogs for the prevention of lymphatic leakage is not recommended. Further RCTs that unify the type and administration of somatostatin analogs are needed to fully understand the effects of somatostatin analogs on the prevention of lymphatic leakage in patients undergoing axillary lymph node dissection for breast cancer.

## Article Information

### Conflicts of Interest

None

### Author Contributions

Study concept: SH and JW; study design: SH, JW, and MS; statistical analyses: SH and JW; interpretation of data: SH and JW; manuscript preparation: SH and JW; manuscript editing: SH, JW, AM, and MS; manuscript review: SH, JW, AM, MS, and NS; and literature screening: SH, JW, and AM. All authors approved the final version and agreed to be accountable for the accuracy and integrity of the work.

### Approval by Institutional Review Board (IRB)

Not applicable.

## Supplement

Supplementary 1～3 Supplementary 1: The electronic database search strategy
Supplementary 2: The trial registry search strategy
Supplementary 3: Risk of bias for the eligibility studies

Appendix Figure 1～4 Appendix Figure 1: Forest plot of A) the volume of drained fluid, B) the duration of drainage, and C) seroma by the somatostatin analog type
Appendix Figure 2: Forest plot of A) the volume of drained fluid, B) the duration of drainage, and C) seroma in only the participants with complete data
Appendix Figure 3: Forest plot of the length of hospital stay by the somatostatin analog type
Appendix Figure 4: Forest plot of A) hematoma, B) surgical site infections, and C) length of hospital stay in only the participants with complete data
