# Supplementary material for: The Efficacy and Safety of Somatostatin Analog after Axillary Node Dissection in Breast Cancer: A Systematic Review and Meta-analysis
Source: JMA J. 2023 May 22;6(3):274–81. doi: 10.31662/jmaj.2022-0219 (PMC10407358; doi:10.31662/jmaj.2022-0219)
Supplement: Supplementary 1～3 — Supplementary 1: The electronic database search strategy Supplementary 2: The trial registry search strategy Supplementary 3: Risk of bias for the eligibility studies [file 2433-3298-6-3-0274-s001.pdf]

Supplementary 1: Electronic database strategy

MEDLINE (PubMed)

1. Breast Neoplasms [Mesh]
2. breast[tiab] AND (cancer*[tiab] OR neoplasm*[tiab] OR carcinoma*[tiab] OR tumor*[tiab])
3. #1 OR #2
4. (“Mastectomy” [Mesh]) OR (“Mastectom*”[tiab]) OR (“Mammectom*”[tiab]) OR (“Breast-Conserving”[tiab]) OR ("axilla" [MeSH] OR "axilla*"[tiab])
5. #3 OR #4
6. Somatostatin[Mesh] OR Octreotide[Mesh] OR lanreotide[Substance Name] OR pasireotide[Substance Name] OR vapreotide [Substance Name] OR somatostatin [tiab] OR octreotide [tiab] OR lanreotide [tiab] OR pasireotide [tiab] OR vapreotide [tiab]
7. #5 AND #6
8. (randomized controlled trial [pt] OR controlled clinical trial [pt] OR randomized [tiab] OR drug therapy[sh] OR placebo [tiab] OR randomly[tiab] OR trial[tiab] OR groups[tiab]) NOT (animals [mh] NOT humans [mh])

9. #7 AND #8

CENTRAL (Cochrane Library)

((([mh "Breast Neoplasms"]) OR (breast:ti,ab AND (cancer*:ti,ab OR neoplasm*:ti,ab OR carcinoma*:ti,ab OR tumor*:ti,ab))) OR (([mh Mastectomy]) OR (Mastectom*:ti,ab) OR (Mammectom*:ti,ab) OR (Breast-Conserving:ti,ab) OR ([mh axilla] OR axilla*:ti,ab))) AND ([mh Somatostatin] OR [mh Octreotide] OR "lanreotide[Substance Name]" OR "pasireotide[Substance Name]" OR "vapreotide [Substance Name]" OR somatostatin:ti,ab OR octreotide:ti,ab OR lanreotide:ti,ab OR pasireotide:ti,ab OR vapreotide:ti,ab)

EMBASE (Dialog)

S1 (EMB.EXACT.EXPLODE("breast tumor"))

S2 EMB.EXACT.EXPLODE("mastectomy") OR (ab(Mastectom*) OR ti(Mastectom*)) OR (ab(Mammectom*) OR ti(Mammectom*)) OR (ab(Breast-Conserving) OR ti(Breast-Conserving)) OR EMB.EXACT.EXPLODE("axilla") OR (ab(axilla*) OR ti(axilla*))

S3 EMB.EXACT.EXPLODE("mastectomy") OR (ab(Mastectom*) OR ti(Mastectom*)) OR (ab(Mammectom*) OR ti(Mammectom*)) OR (ab(Breast-Conserving) OR ti(Breast-Conserving)) OR EMB.EXACT.EXPLODE("axilla") OR (ab(axilla*) OR ti(axilla*))

S4 S1 OR S2 OR S3

S5 EMB.EXACT.EXPLODE("somatostatin") OR EMB.EXACT.EXPLODE("octreotide") OR (ab(somatostatin) OR ti(somatostatin)) OR (ab(octreotide) OR ti(octreotide)) OR (ab(lanreotide) OR ti(lanreotide)) OR (ab(pasireotide) OR ti(pasireotide)) OR (ab(vapreotide) OR ti(vapreotide))

S6 S4 AND S5

S7 (ab(random*) OR ti(random*)) OR (ab(placebo*) OR ti(placebo*)) OR (ab(double NEAR/1 blind*) OR ti(double NEAR/1 blind*))

S8 S6 AND S7

Supplementary 2: Clinical trial registry

ICTRP

(breast OR mastectomy OR axilla OR axillary) AND (somatostatin OR octreotide OR lanreotide OR pasireotide OR vapreotide)

ClinicalTrials.gov

Condition or disease: (breast OR mastectomy OR axilla OR axillary)

Intervention: (somatostatin OR octreotide OR lanreotide OR pasireotide OR vapreotide)

Supplementary 3: Quality scores for the eligibility studies

The duration of drainage

Authors [ref no.]	Risk of bias 2 tool assessment					
	Bias arising from the randomization process	Bias due to deviations from intended interventions	Bias due to missing outcome data	Bias in measurement of the outcome	Bias in selection of the reported results	Overall risk of bias
Carcoforo [17]	Some concerns	Low	Low	Low	Some concerns	Some concerns
Mahmoud [18]	Some concerns	Low	Low	Some concerns	Some concerns	Some concerns
Chemi SpA [19]	Some concerns	Low	Low	Low	Low	Some concerns
Gauthier [20]	Some concerns	Low	Low	Low	Some concerns	Some concerns
Chéreau [21]	Low	Low	Low	Low	Low	Low
Prajapati [23]	Low	Low	Low	Low	Low	Low

Seroma

Authors [ref no.]	Risk of bias 2 tool assessment					
	Bias arising from the randomization process	Bias due to deviations from intended interventions	Bias due to missing outcome data	Bias in measurement of the outcome	Bias in selection of the reported results	Overall risk of bias
Gauthier [20]	Some concerns	Low	Low	Low	Some concerns	Some concerns
Chéreau [21]	Low	Low	Low	Low	Low	Low
Chemi SpA [22]	Some concerns	Low	Low	Low	Low	Some concerns
Prajapati [23]	Low	Low	Low	Low	Low	Low

Hematoma

Authors [ref no.]	Risk of bias 2 tool assessment					
	Bias arising from the randomization process	Bias due to deviations from intended interventions	Bias due to missing outcome data	Bias in measurement of the outcome	Bias in selection of the reported results	Overall risk of bias
Carcoforo [17]	Some concerns	Low	Low	Low	Some concerns	Some concerns
Chemi SpA [19]	Some concerns	Low	Low	Low	Some concerns	Some concerns
Chéreau [21]	Low	Low	Low	Low	Low	Low

Surgical site infection

Authors [ref no.]	Risk of bias 2 tool assessment					
	Bias arising from the randomizati	Bias due to deviations from	Bias due to missing outcome	Bias in measuremen t of the	Bias in selection of the reported	Overall risk of bias

	on process	intended interventions	data	outcome	results	
Carcoforo [17]	Some concerns	Low	Low	Low	Some concerns	Some concerns
Chemi SpA [19]	Some concerns	Low	Low	Low	Some concerns	Some concerns
Gauthier [20]	Some concerns	Low	Low	Low	Some concerns	Some concerns
Chemi SpA [22]	Some concerns	Low	Low	Low	Some concerns	Some concerns
Prajapati [23]	Low	Low	Low	Low	Low	Low

Length of the hospital stay

Authors [ref no.]	Risk of bias 2 tool assessment					
	Bias arising from the randomization process	Bias due to deviations from intended interventions	Bias due to missing outcome data	Bias in measurement of the outcome	Bias in selection of the reported results	Overall risk of bias
Carcoforo [17]	Some concerns	Low	Low	Some concerns	Some concerns	Some concerns
Gauthier [20]	Some concerns	Low	Low	Low	Some concerns	Some concerns
Chéreau [21]	Low	Low	Low	Low	Low	Low
Prajapati [23]	Low	Low	Low	Some concerns	Low	Some concerns

All adverse events

Authors [ref no.]	Risk of bias 2 tool assessment					
	Bias arising from the randomization process	Bias due to deviations from intended interventions	Bias due to missing outcome data	Bias in measurement of the outcome	Bias in selection of the reported results	Overall risk of bias

Chemi SpA [19]	Some concerns	Low	Low	Low	Some concerns	Some concerns
Chéreau [21]	Low	Low	Low	Low	Low	Low
Chemi SpA [22]	Some concerns	Low	Low	Low	Some concerns	Some concerns
Prajapati [23]	Low	Low	Low	Low	Low	Low
