# Supplementary material for: The Efficacy and Safety of Somatostatin Analog after Axillary Node Dissection in Breast Cancer: A Systematic Review and Meta-analysis
Source: JMA J. 2023 May 22;6(3):274–81. doi: 10.31662/jmaj.2022-0219 (PMC10407358; doi:10.31662/jmaj.2022-0219)

Appendix Figure 1A

	Somatostatin analogue			Control				Mean Difference	
**Study or Subgroup**	**Mean**	**SD**	**Total**	**Mean**	**SD**	**Total**	**Weight**	**IV, Random, 95% CI**	**Year**
**3.1.1 octreotide**									
Carcoforo 2003	65.4	21.1	125	94.6	19	136	43.6%	-29.20 [-34.09, -24.31]	2003
Chemi SpA 2012	104	29	30	145	45.8	20	28.5%	-41.00 [-63.60, -18.40]	2012
Prajapati 2021	105.28	51.83	15	92.43	46.83	15	18.7%	12.85 [-22.50, 48.20]	2021
**Subtotal (95% CI)**			**170**			**171**	**90.8%**	**-24.37 [-45.00, -3.74]**	
Heterogeneity: Tau² = 221.96; Chi² = 6.47, df = 2 (P = 0.04); I² = 69%									
Test for overall effect: Z = 2.32 (P = 0.02)									
**3.1.2 pasireotide**									
Chéreau 2016	297.5	181.37	42	255.5	240.81	48	4.7%	42.00 [-45.46, 129.46]	2016
**Subtotal (95% CI)**			**42**			**48**	**4.7%**	**42.00 [-45.46, 129.46]**	
Heterogeneity: Not applicable									
Test for overall effect: Z = 0.94 (P = 0.35)									
**3.1.3 lanreotide**									
Gauthier 2012	292	241	72	337	307.5	73	4.5%	-45.00 [-134.86, 44.86]	2012
**Subtotal (95% CI)**			**72**			**73**	**4.5%**	**-45.00 [-134.86, 44.86]**	
Heterogeneity: Not applicable									
Test for overall effect: Z = 0.98 (P = 0.33)									
**Total (95% CI)**			**284**			**292**	**100.0%**	**-22.07 [-42.09, -2.05]**	
Heterogeneity: Tau² = 232.87; Chi² = 9.12, df = 4 (P = 0.06); I² = 56%									
Test for overall effect: Z = 2.16 (P = 0.03)									
Test for subgroup differences: Chi² = 2.36, df = 2 (P = 0.31), I² = 15.2%									

Mean Difference
IV, Random, 95% CI

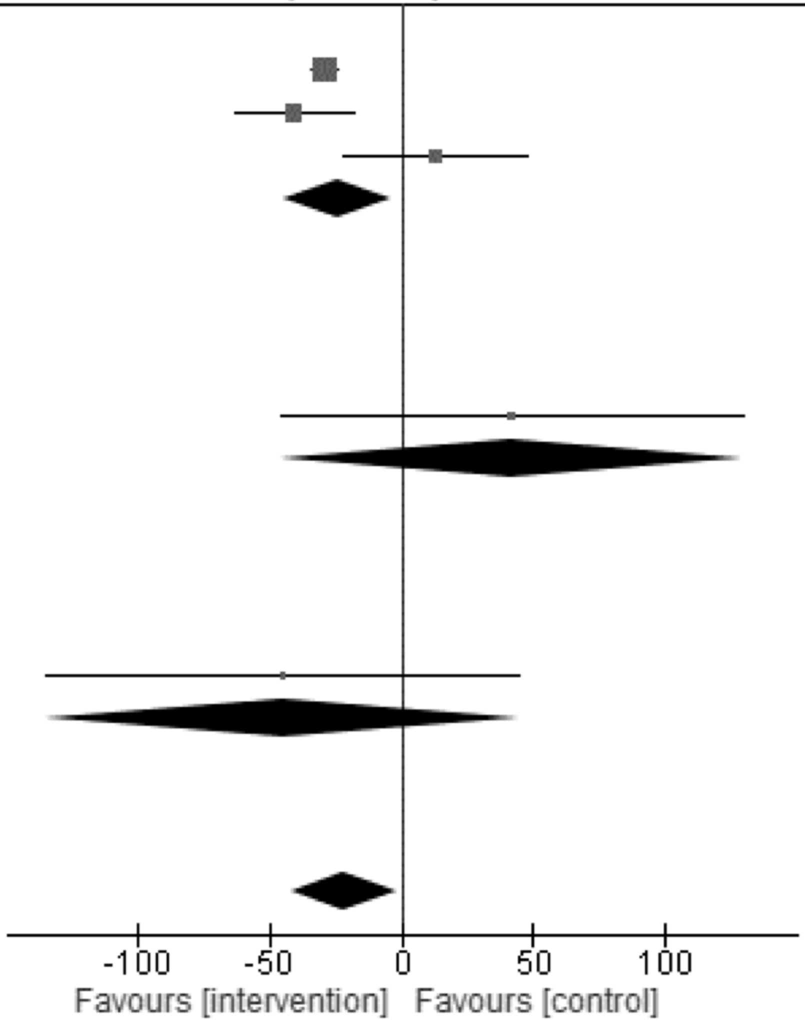

Appendix Figure 1B

Study or Subgroup	Somatostatin analogue Mean	SD	Total	Control Mean	SD	Total	Weight	Mean Difference IV, Random, 95% CI	Year
**3.2.1 octreotide**									
Carcoforo 2003	4.2	1.9	125	3.9	1.5	136	23.8%	0.30 [-0.12, 0.72]	2003
Mahmoud 2007	12.7	6.74	30	25	9.57	20	3.3%	-12.30 [-17.14, -7.46]	2007
Chemi SpA 2012	9.1667	8.3309	81	7	2.7	28	11.4%	2.17 [0.10, 4.24]	2012
Prajapati 2021	3.13	1.36	15	4.93	2.49	15	15.9%	-1.80 [-3.24, -0.36]	2021
**Subtotal (95% CI)**			**251**			**199**	**54.4%**	**-1.77 [-4.49, 0.96]**	
Heterogeneity: $Tau^2 = 6.31$; $Chi^2 = 36.65$, df = 3 ($P < 0.00001$); $I^2 = 92\%$									
Test for overall effect: Z = 1.27 (P = 0.20)									
**3.2.2 pasireotide**									
Chéreau 2016	3	1.11	42	3	0.74	48	24.0%	0.00 [-0.40, 0.40]	2016
**Subtotal (95% CI)**			**42**			**48**	**24.0%**	**0.00 [-0.40, 0.40]**	
Heterogeneity: Not applicable									
Test for overall effect: Z = 0.00 (P = 1.00)									
**3.2.3 lanreotide**									
Gauthier 2012	4	1.75	72	4.5	2.75	73	21.6%	-0.50 [-1.25, 0.25]	2012
**Subtotal (95% CI)**			**72**			**73**	**21.6%**	**-0.50 [-1.25, 0.25]**	
Heterogeneity: Not applicable									
Test for overall effect: Z = 1.31 (P = 0.19)									
**Total (95% CI)**			**365**			**320**	**100.0%**	**-0.48 [-1.43, 0.46]**	
Heterogeneity: $Tau^2 = 0.93$; $Chi^2 = 38.76$, df = 5 ($P < 0.00001$); $I^2 = 87\%$									
Test for overall effect: Z = 1.00 (P = 0.32)									
Test for subgroup differences: $Chi^2 = 2.74$, df = 2 (P = 0.25), $I^2 = 26.9\%$									

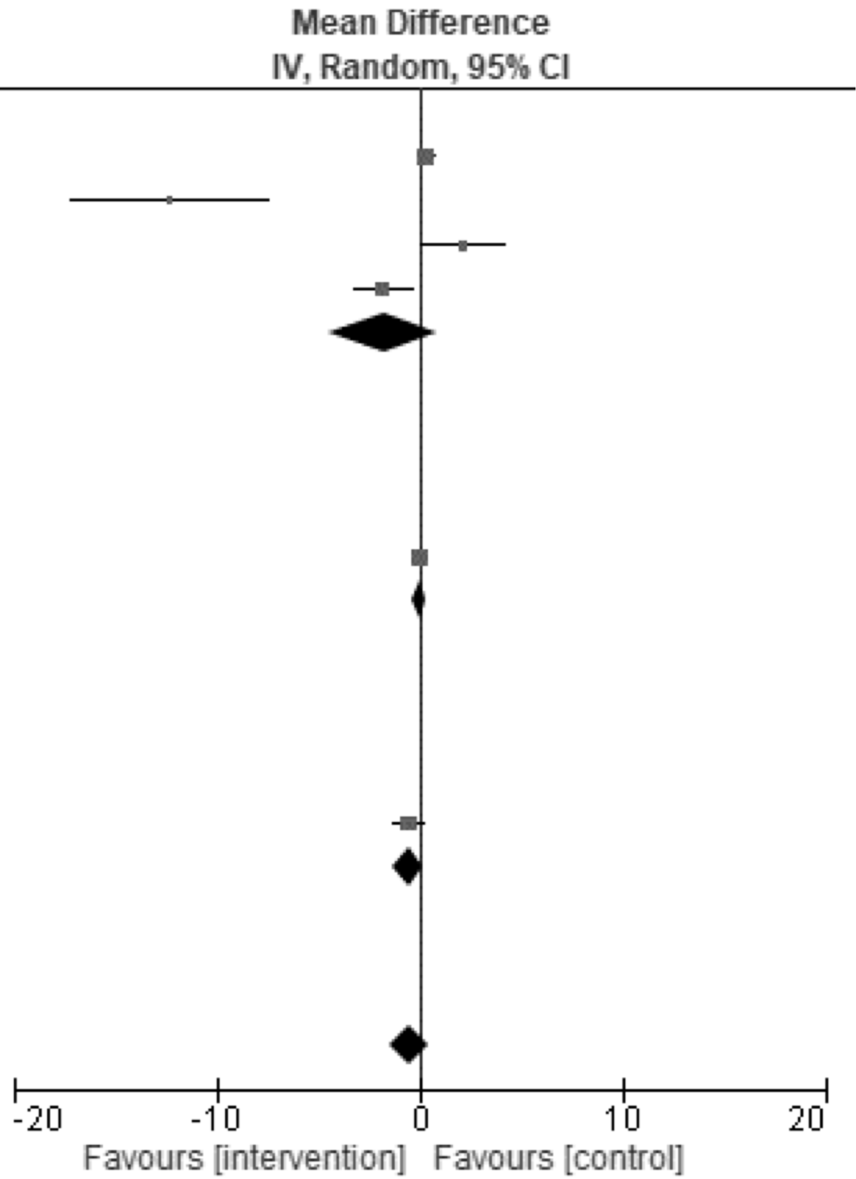

Appendix Figure 1C

	Somatostatin analogue		Control			Risk Ratio	
**Study or Subgroup**	**Events**	**Total**	**Events**	**Total**	**Weight**	**M-H, Random, 95% CI**	**Year**
**3.3.1 octreotide**							
Chemi SpA 2020	18	23	15	23	34.2%	1.20 [0.83, 1.73]	2020
Prajapati 2021	2	15	9	15	7.1%	0.22 [0.06, 0.86]	2021
**Subtotal (95% CI)**		**38**		**38**	**41.3%**	**0.57 [0.09, 3.81]**	
Total events	20		24				
Heterogeneity: Tau² = 1.65; Chi² = 7.43, df = 1 (P = 0.006); I² = 87%							
Test for overall effect: Z = 0.58 (P = 0.56)							
**3.3.2 pasireotide**							
Chéreau 2016	17	42	21	48	27.9%	0.93 [0.57, 1.51]	2016
**Subtotal (95% CI)**		**42**		**48**	**27.9%**	**0.93 [0.57, 1.51]**	
Total events	17		21				
Heterogeneity: Not applicable							
Test for overall effect: Z = 0.31 (P = 0.75)							
**3.3.3 lanreotide**							
Gauthier 2012	25	72	28	73	30.8%	0.91 [0.59, 1.39]	2012
**Subtotal (95% CI)**		**72**		**73**	**30.8%**	**0.91 [0.59, 1.39]**	
Total events	25		28				
Heterogeneity: Not applicable							
Test for overall effect: Z = 0.45 (P = 0.65)							
**Total (95% CI)**		**152**		**159**	**100.0%**	**0.91 [0.61, 1.34]**	
Total events	62		73				
Heterogeneity: Tau² = 0.08; Chi² = 6.64, df = 3 (P = 0.08); I² = 55%							
Test for overall effect: Z = 0.49 (P = 0.63)							
Test for subgroup differences: Chi² = 0.24, df = 2 (P = 0.89), I² = 0%							

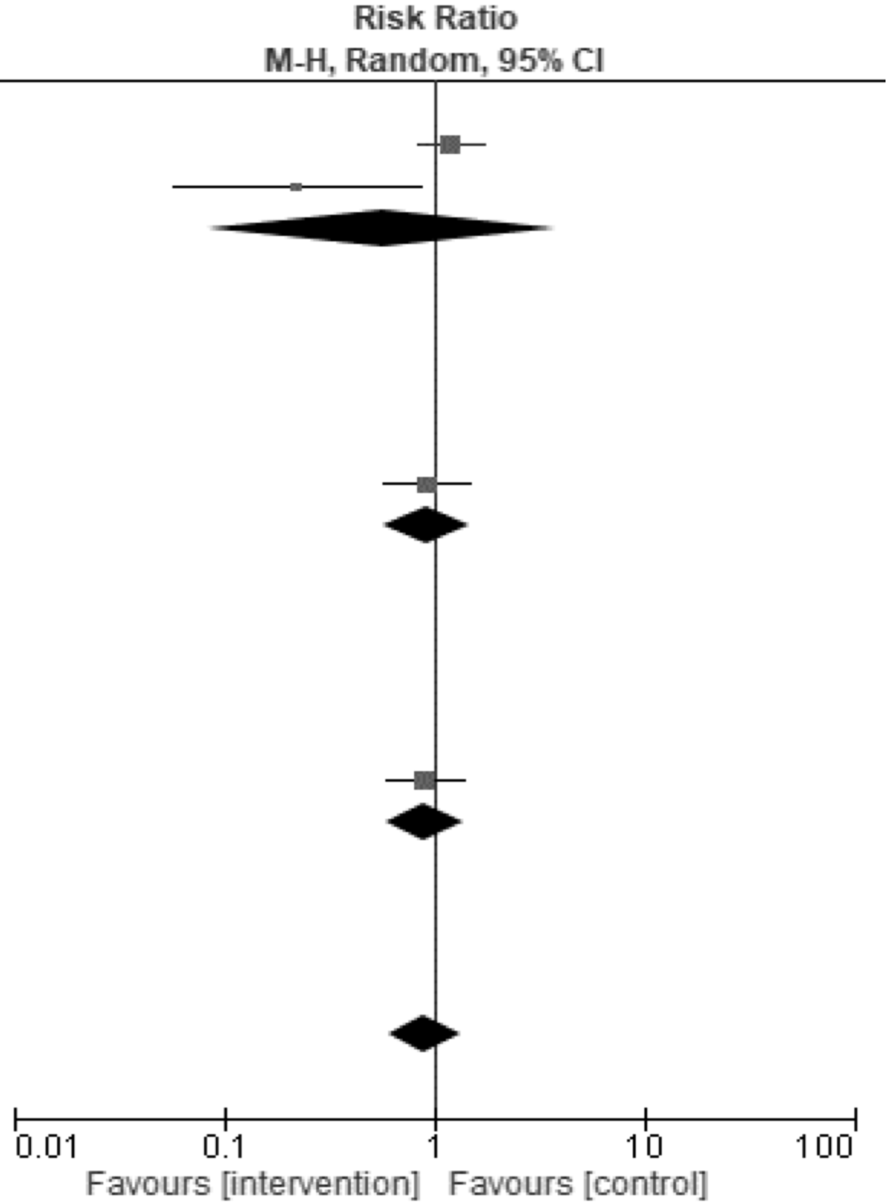

Appendix Figure 2A

Study or Subgroup	Somatostatin analogue Mean	SD	Total	Control Mean	SD	Total	Weight	Mean Difference IV, Random, 95% CI	Year
Carcoforo 2003	65.4	21.1	125	94.6	19	136	59.0%	-29.20 [-34.09, -24.31]	2003
Prajapati 2021	105.28	51.83	15	92.43	46.83	15	41.0%	12.85 [-22.50, 48.20]	2021
**Total (95% CI)**			**140**			**151**	**100.0%**	**-11.97 [-52.50, 28.56]**	

Heterogeneity: Tau² = 718.35; Chi² = 5.33, df = 1 (P = 0.02); I² = 81%

Test for overall effect: Z = 0.58 (P = 0.56)

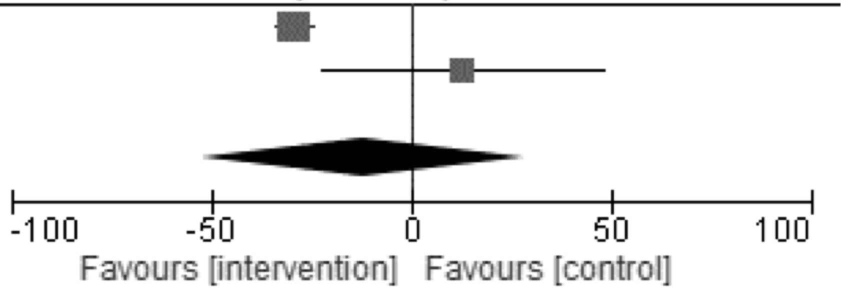

Appendix Figure 2B

Study or Subgroup	Somatostatin analogue Mean	SD	Total	Control Mean	SD	Total	Weight	Mean Difference IV, Random, 95% CI	Year
Carcoforo 2003	4.2	1.9	125	3.9	1.5	136	41.2%	0.30 [-0.12, 0.72]	2003
Chemi SpA 2012	9.1667	8.3309	81	7	2.7	28	26.2%	2.17 [0.10, 4.24]	2012
Prajapati 2021	3.13	1.36	15	4.93	2.49	15	32.6%	-1.80 [-3.24, -0.36]	2021
**Total (95% CI)**			**221**			**179**	**100.0%**	**0.10 [-1.61, 1.82]**	

Heterogeneity: Tau² = 1.81; Chi² = 11.13, df = 2 (P = 0.004); I² = 82%

Test for overall effect: Z = 0.12 (P = 0.91)

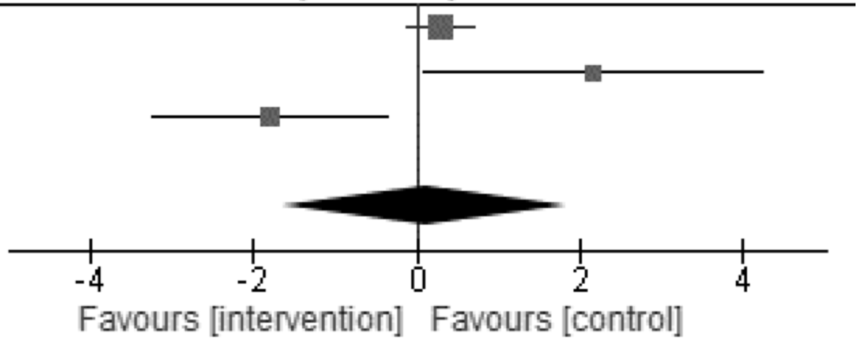

Appendix Figure 2C

Study or Subgroup	Somatostatin analogue Events	Total	Control Events	Total	Weight	Risk Ratio M-H, Random, 95% CI	Year
Chemi SpA 2020	18	23	15	23	55.8%	1.20 [0.83, 1.73]	2020
Prajapati 2021	2	15	9	15	44.2%	0.22 [0.06, 0.86]	2021
**Total (95% CI)**		**38**		**38**	**100.0%**	**0.57 [0.09, 3.81]**	
Total events	20		24				

Heterogeneity: Tau² = 1.65; Chi² = 7.43, df = 1 (P = 0.006); I² = 87%

Test for overall effect: Z = 0.58 (P = 0.56)

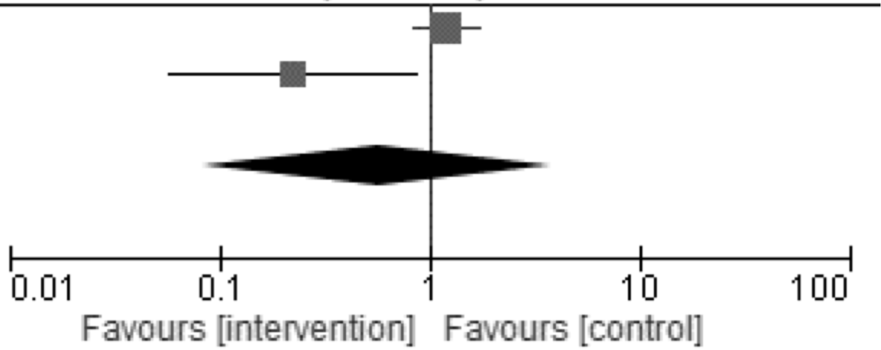

Appendix Figure 3

	Somatostatin analogue			Control				Mean Difference	
Study or Subgroup	Mean	SD	Total	Mean	SD	Total	Weight	IV, Random, 95% CI	Year
**3.4.1 octreotide**									
Carcoforo 2003	5.13	1.06	15	7.06	2.4	15	11.1%	-1.93 [-3.26, -0.60]	2003
Prajapati 2021	4.7	2.1	125	5	2.3	136	29.3%	-0.30 [-0.83, 0.23]	2021
**Subtotal (95% CI)**			**140**			**151**	**40.4%**	**-1.00 [-2.58, 0.58]**	
Heterogeneity: Tau² = 1.06; Chi² = 4.98, df = 1 (P = 0.03); I² = 80%									
Test for overall effect: Z = 1.24 (P = 0.22)									
**3.4.2 pasireotide**									
Chéreau 2016	4	1.75	72	4.5	2.75	73	22.4%	-0.50 [-1.25, 0.25]	2016
**Subtotal (95% CI)**			**72**			**73**	**22.4%**	**-0.50 [-1.25, 0.25]**	
Heterogeneity: Not applicable									
Test for overall effect: Z = 1.31 (P = 0.19)									
**3.4.3 lanreotide**									
Gauthier 2012	5	0.74	42	6	0.74	48	37.2%	-1.00 [-1.31, -0.69]	2012
**Subtotal (95% CI)**			**42**			**48**	**37.2%**	**-1.00 [-1.31, -0.69]**	
Heterogeneity: Not applicable									
Test for overall effect: Z = 6.40 (P < 0.00001)									
**Total (95% CI)**			**254**			**272**	**100.0%**	**-0.79 [-1.30, -0.27]**	
Heterogeneity: Tau² = 0.16; Chi² = 8.35, df = 3 (P = 0.04); I² = 64%									
Test for overall effect: Z = 3.00 (P = 0.003)									
Test for subgroup differences: Chi² = 1.47, df = 2 (P = 0.48), I² = 0%									

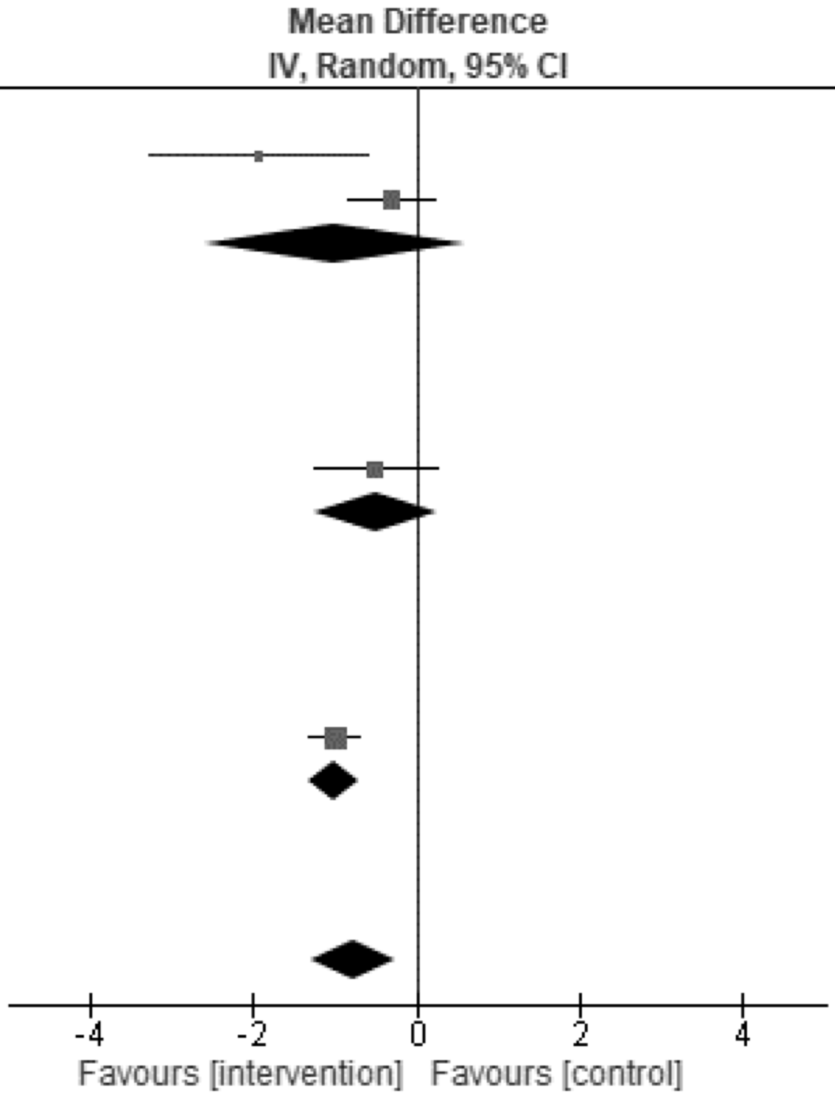

Appendix Figure 4A

Study or Subgroup	Somatostatin analogue Events	Somatostatin analogue Total	Control Events	Control Total	Weight	Risk Ratio M-H, Random, 95% CI	Year
Carcoforo 2003	0	125	1	136	35.4%	0.36 [0.01, 8.82]	2003
Chemi SpA 2012	2	86	1	28	64.6%	0.65 [0.06, 6.91]	2012
**Total (95% CI)**		**211**		**164**	**100.0%**	**0.53 [0.08, 3.53]**	
Total events	2		2				

Heterogeneity: Tau² = 0.00; Chi² = 0.09, df = 1 (P = 0.77); I² = 0%

Test for overall effect: Z = 0.66 (P = 0.51)

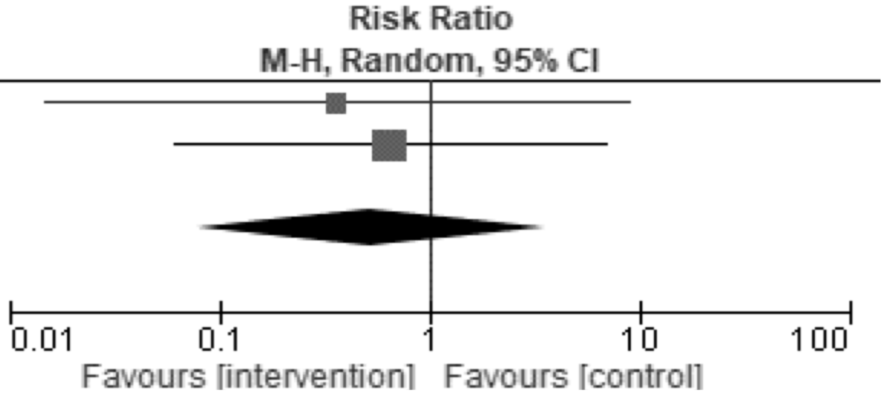

Appendix Figure 4B

Study or Subgroup	Somatostatin analogue Events	Somatostatin analogue Total	Control Events	Control Total	Weight	Risk Ratio M-H, Random, 95% CI	Year
Carcoforo 2003	1	125	2	136	30.2%	0.54 [0.05, 5.93]	2003
Chemi SpA 2012	2	86	1	28	30.8%	0.65 [0.06, 6.91]	2012
Chemi SpA 2020	2	24	0	24	19.3%	5.00 [0.25, 98.96]	2020
Prajapati 2021	0	15	2	15	19.7%	0.20 [0.01, 3.85]	2021
**Total (95% CI)**		**250**		**203**	**100.0%**	**0.72 [0.20, 2.69]**	
Total events	5		5				

Heterogeneity: Tau² = 0.00; Chi² = 2.41, df = 3 (P = 0.49); I² = 0%

Test for overall effect: Z = 0.48 (P = 0.63)

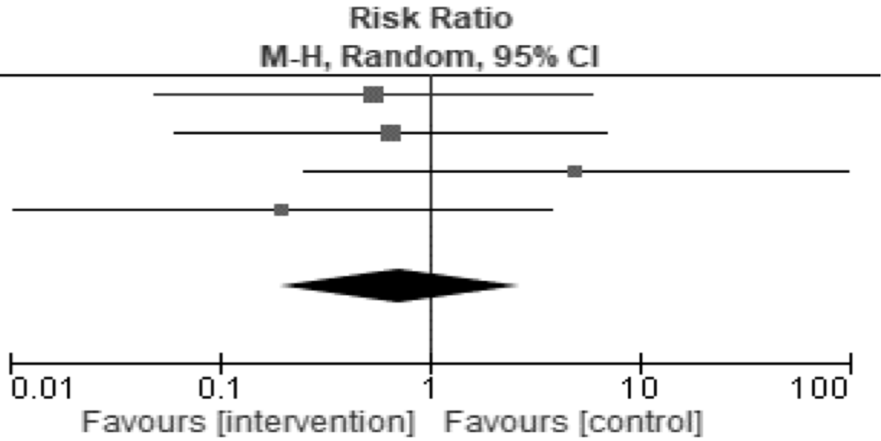

Appendix Figure 4C

Study or Subgroup	Somatostatin analogue Mean	Somatostatin analogue SD	Somatostatin analogue Total	Control Mean	Control SD	Control Total	Weight	Mean Difference IV, Random, 95% CI	Year
Carcoforo 2003	5.13	1.06	15	7.06	2.4	15	42.8%	-1.93 [-3.26, -0.60]	2003
Prajapati 2021	4.7	2.1	125	5	2.3	136	57.2%	-0.30 [-0.83, 0.23]	2021
**Total (95% CI)**			**140**			**151**	**100.0%**	**-1.00 [-2.58, 0.58]**	

Heterogeneity: Tau² = 1.06; Chi² = 4.98, df = 1 (P = 0.03); I² = 80%

Test for overall effect: Z = 1.24 (P = 0.22)

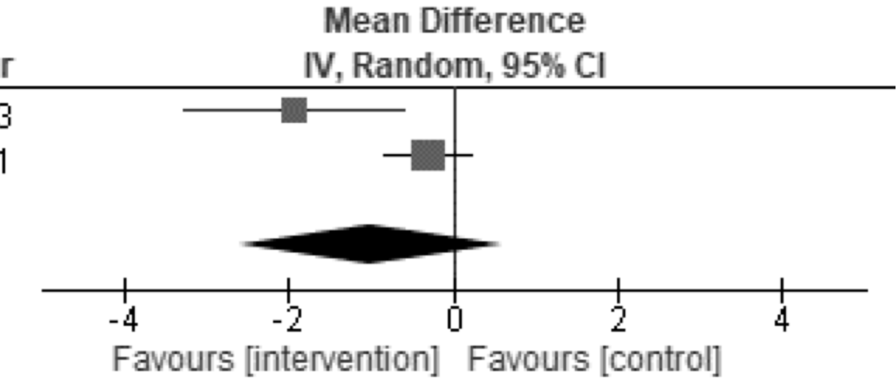

Supplement: Appendix Figure 1～4 — Appendix Figure 1: Forest plot of A) the volume of drained fluid, B) the duration of drainage, and C) seroma by the somatostatin analog type Appendix Figure 2: Forest plot of A) the volume of drained fluid, B) the duration of drainage, and C) seroma in only the participants with complete data Appendix Figure 3: Forest plot of the length of hospital stay by the somatostatin analog type Appendix Figure 4: Forest plot of A) hematoma, B) surgical site infections, and C) length of hospital stay in only the participants with complete data [file 2433-3298-6-3-0274-s002.pdf]
